# Combining Physio-Biochemical Characterization and Transcriptome Analysis Reveal the Responses to Varying Degrees of Drought Stress in *Brassica napus* L.

**DOI:** 10.3390/ijms23158555

**Published:** 2022-08-02

**Authors:** Shuai Fang, Peimin Zhao, Zengdong Tan, Yan Peng, Lintang Xu, Yutong Jin, Fang Wei, Liang Guo, Xuan Yao

**Affiliations:** 1 National Key Laboratory of Crop Genetic Improvement, Huazhong Agricultural University, Wuhan 430070, China; fangshuai19940703@webmail.hzau.edu.cn (S.F.); zhaopeimin@webmail.hzau.edu.cn (P.Z.); zdtan@webmail.hzau.edu.cn (Z.T.); pengyan0927@gmail.com (Y.P.); xulint@webmail.hzau.edu.cn (L.X.); jinyt@webmail.hzau.edu.cn (Y.J.); guoliang@mail.hzau.edu.cn (L.G.); 2 Hubei Hongshan Laboratory, Wuhan 430070, China; 3 Shenzhen Branch, Guangdong Laboratory for Lingnan Modern Agriculture, Genome Analysis Laboratory of the Ministry of Agriculture, Agricultural Genomics Institute at Shenzhen, Chinese Academy of Agricultural Sciences, Shenzhen 518000, China; 4 Oil Crops Research Institute of the Chinese Academy of Agricultural Sciences, Key Laboratory of Oilseeds Processing of Ministry of Agriculture and Hubei Key Laboratory of Lipid Chemistry and Nutrition, Wuhan 430062, China; weifang@caas.cn; 5 Shenzhen Institute of Nutrition and Health, Huazhong Agricultural University, Wuhan 430070, China

**Keywords:** *Brassica napus*, physio-biochemical characterization, transcriptome, drought tolerance, *BnaCIPK6*

## Abstract

*Brassica napus* L. has become one of the most important oil-bearing crops, and drought stress severely influences its yield and quality. By combining physio-biochemical characterization and transcriptome analysis, we studied the response of *B. napus* plants to different degrees of drought stress. Some physio-biochemical traits, such as fresh weight (FW), dry weight (DW), abscisic acid (ABA) content, net photosynthetic rate (Pn), stomatal conductance (g_s_), and transpiration rate (Tr), were measured, and the total content of the epidermal wax/cutin, as well as their compositions, was determined. The results suggest that both stomatal transpiration and cuticular transpiration are affected when *B. napus* plants are subjected to varying degrees of drought stress. A total of 795 up-regulated genes and 1050 down-regulated genes were identified under severe drought stress by transcriptome analysis. Gene ontology (GO) enrichment analysis of differentially expressed genes (DEGs) revealed that the up-regulated genes were mainly enriched in the stress response processes, such as response to water deprivation and abscisic acid, while the down-regulated genes were mainly enriched in the chloroplast-related parts affecting photosynthesis. Moreover, overexpression of *BnaA01.CIPK6,* an up-regulated DEG, was found to confer drought tolerance in *B. napus*. Our study lays a foundation for a better understanding of the molecular mechanisms underlying drought tolerance in *B. napus*.

## 1. Introduction

Drought stress has a serious impact on agricultural production. Over the past decade, drought has cost the world USD 30 billion in crop production losses [1]. Along with the global warming, the frequency of drought stress is increasing, and fresh water supplies for irrigation are decreasing. As sessile organisms, plants must adapt to the changes of the external environment; they thus evolve a variety of mechanisms to cope with drought stress [2]. *B. napus* has become one of the most important agricultural crop species worldwide, providing edible oil, animal feed, and biodiesel. However, its growth is always susceptible to seasonal or persistent drought stress. It is therefore urgent to understand the mechanism of drought resistance and to breed drought-resistant varieties in *B. napus*.

In general, there are two main ways in which plants regulate water loss: stomatal transpiration and cuticular transpiration [3]. Stomatal transpiration is the more important one for water loss, and cuticle transpiration only accounts for 5–10% of plant water loss under normal conditions [4]. However, cuticular transpiration becomes the major route for water loss after stomatal closure when the plants have suffered from drought stress [5]. The drought damage to a plant is multi-faceted, including the effects on its shape, the physiological damage, and widespread changes in cellular processes. When plants are subjected to drought stress, phytohormones such as abscisic acid (ABA) are synthesized [6], and the content and composition of the cuticle are also altered [7]. These changes in the physio-biochemical processes make plants adapt to drought stress through the regulation of water loss in these two ways and allow plants to complete their growth cycle rapidly. ABA induced by drought stress can trigger stomatal closure and then, consequently, affect plant photosynthesis [8,9,10]. The negative effects of drought stress on plant photosynthesis include reduced stomatal conductance and transpiration rate [11]. The biomass is greatly reduced as a result of defects in growth and development under drought stress. The genes involved in the ABA biosynthesis pathway and the signal transduction pathway, such as *NCED3* [12,13], *PYL9* [14], *PYL10* [15], *PP2C-As* [16], and *SnRK2.2/2.3/2.6* [17], have been reported to be closely related to drought tolerance in plants. The pyrabactin resistance/pyrabactin resistance 1-like/regulatory components of the ABA receptor (PYR/PYL/RCAR) protein family have been identified to be ABA receptors [18,19]. Under normal conditions, the activity of sucrose non-fermenting 1-related protein kinase 2 (SnRK2s) is inhibited by active clade A protein phosphatase type 2C (PP2C). In the presence of ABA, the receptor binds to ABA and PP2C. The inhibition of SnRK2s by PP2C is then relieved. SnRK2s are further activated, and they can directly phosphorylate and activate transcription factors which regulate downstream stress-resistant gene expression such as abscisic acid-responsive element-binding transcription factors 3 (*ABF3*).

Cuticular transpiration is also an important pathway for water loss, which is controlled by the cuticle [20]. The cuticle is an extracellular lipid structure formed by cutin and epidermal wax deposited on the epidermal cell wall from inside to outside. It can seal the plant epidermis and protect it from biotic and abiotic stresses [21]. Plants respond and adapt to drought stress by increasing cuticle content and changing its composition [22]. The first step of cuticle synthesis is the de novo synthesis of fatty acids. After being synthesized in plastid, the C16:0-ACP and C18:0-ACP precursors are first removed by acyl-ACP thioesterase, and then, the acyl group is transferred to CoA catalyzed by long chain acyl-CoA synthase (*LACS*) for the synthesis of Acyl-CoAs [23]. Acyl-CoAs act as substrates for the synthesis of very long chain acyl-CoAs (VLC acyl-CoAs). This reaction is catalyzed by the fatty acid elongase complex (*FAE*) located in the endoplasmic reticulum. Wax synthesis is divided into two pathways: the alkane synthesis pathway and the primary alcohol synthesis pathway; these account for about 20% and 80% of the stem wax synthesis in Arabidopsis, respectively. Alkanes, aldehydes, secondary alcohols, and ketones are synthesized by the alkane pathway, while primary alcohols and esters are synthesized by the primary alcohol synthesis pathway. ABC transporters are responsible for the transport of these waxy substances from the plasma membrane to the exoplasm. Subsequently, the trans-cell wall transport from the extoplasm to the plant epidermis can be realized in two different ways; one is through lipid transfer proteins (LTPs), and the other is through the local hydrophobic zone formed by proteins and sugars in the cell wall [24]. For the cutin synthesis, after the cutin monomers are synthesized by fatty acid activation, epoxidation, and hydroxylation they then undergo polyesterification to form cutin polyester [25]. The cytochrome P450 enzymes catalyze the conversion of the long-chain fatty acids into long-chain hydroxylated and epoxidated fatty acids. Glycerol-3-phosphate acyltransferases such as *GPAT6* are responsible for the reaction from the cutin monomer to monoacylglycerol [26]. Monoacylglycerol monomers exit the cell through specific ABC transporters in the plasma membrane, and LTPs also participate in the transport of cutin [27]. Recent research shows that changes in the cutin monomer composition and epidermal wax hydrophobicity were all involved in the drought resistance of sorghums [28].

Intensive studies have focused on the responses of plants to drought stress; however, the changes in the physio-biochemical processes and gene expression of *B. napus* plants subjected to varying degrees of drought stress remains unclear. In this study, we analyzed the physio-biochemical traits under varying degrees of drought stress and identified DEGs in response to severe drought stress by RNA sequencing (RNA-seq) analysis in *B. napus*. Moreover, the function of *BnaCIPK6*, an up-regulated gene under severe drought stress, was further validated. Our results revealed that stomatal transpiration and cuticular transpiration play vital roles in the response to varying degrees of drought stress in *B. napus*. This study will improve the understanding of molecular mechanisms underlying the adaptation to drought stress in *B. napus*.

## 2. Results

### 2.1. Characterization of Physio-Biochemical Traits at Seedling Stage under Varying Degrees of Drought Stress in B. napus

**Figure 1:**
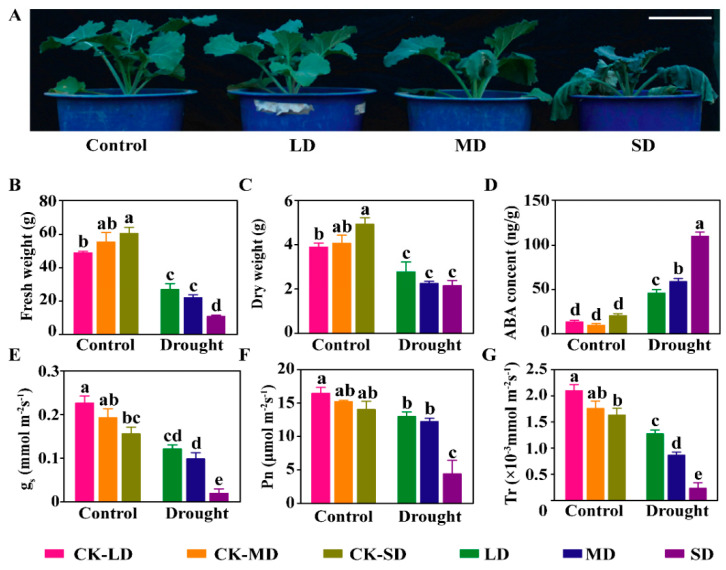
Characterization of physio-biochemical traits responding to varying degrees of drought stress. (**A**) The response of *B. napus* to varying degrees of drought stress. Bar = 10 cm. (**B**) Aboveground fresh weight under varying degrees of drought stress. (**C**) Aboveground dry weight under varying degrees of drought stress. The data are the means ± standard deviation (*n* = 4). (**D**–**G**) Abscisic acid content (**D**), stomatal conductance (**E**), net photosynthetic rate (**F**), transpiration rate (**G**) under varying degrees of drought stress. LD, mild drought stress; MD, moderate drought stress; SD, severe drought stress; CK-LD, CK-MD, and CK-SD, the respective controls of LD, MD and SD. The data are the means ± standard deviation (*n* = 4). Different lowercase letters indicate significant differences, while the same letters indicate no significant difference (one–way ANOVA for multiple comparisons, *p* < 0.05).

In order to evaluate the response of the *B. napus* to varying degrees of drought stress, we determined some physio-biochemical traits at the seedling stage under mild drought stress (LD, Relative Water Content: 50%), moderate drought stress (MD, Relative Water Content: 40%), and severe drought stress (SD, Relative Water Content: 30%), respectively. We set up respective controls under normal conditions (Relative Water Content: 80%) to rule out the effects of growth, named as CK-LD, CK-MD, and CK-SD, corresponding to LD, MD, and SD, respectively. Unlike the response of the seedlings under normal or LD conditions, some leaves of the seedlings under the MD conditions began to wilt, and almost all the leaves became wilted under the SD conditions (Figure 1A). To study the cause of the response variation, the aboveground fresh weight and dry weight were measured at the seedling stage. Due to the different growth periods, both the dry weight and the fresh weight showed a significant increase under the CK-SD conditions compared with CK-LD. Compared with the respective controls, the fresh weight of the seedlings under the LD, MD, and SD conditions decreased by 44.2%, 60.2%, and 82.2%, respectively, and the dry weight of the seedlings decreased by 28.8%, 44.5%, and 56.2%, respectively. Under the SD conditions, the fresh weight of the seedlings decreased by 59.9% and 51.2% compared with the LD and MD conditions, respectively, while there was no significant difference in the dry weight (Figure 1B,C).

Drought stress triggers abscisic acid (ABA) biosynthesis, resulting in ABA accumulation, and finally affects photosynthesis. We measured the ABA content and the parameters related to photosynthesis. There was no significant change in the ABA content between the CK-LD, CK-MD, and CK-SD conditions. However, the ABA contents under the LD, MD, and SD conditions were 2.45 times, 5.23 times, and 4.42 times higher than that under the CK-LD, CK-MD, and CK-SD conditions, respectively (Figure 1D). In order to analyze the effect of the increased ABA content on photosynthesis, we measured the photosynthesis-related parameters. Our results showed that stomatal conductance (g_s_) significantly decreased by 46.3%, 48.9%, and 87.2% under the LD, MD, and SD conditions compared with the CK-LD, CK-MD, and CK-SD conditions, respectively, and significantly decreased by 31.1% under the CK-SD conditions compared with the CK-LD conditions (Figure 1E). The photosynthetic rates (Pn) did not change significantly between the CK-LD, CK-MD, and CK-SD conditions but significantly decreased by 21.1%, 19.5%, and 68.6% under the LD, MD, and SD conditions, respectively (Figure 1F). Similarly, the transpiration rate (Tr) significantly decreased by 39.3%, 50.8%, and 85.8% under the LD, MD, and SD conditions compared with the CK-LD, CK-MD, and CK-SD conditions, respectively (Figure 1G). In addition, the stomatal conductance, photosynthetic rate, and transpiration rate under the SD conditions significantly reduced compared with that under the LD and MD conditions (Figure 1E–G). These results suggest that varying degrees of drought stress cause a series of physio-biochemical responses, and the drought-induced ABA may promote stomatal closure, which in turn limits water loss through transpiration and affects photosynthesis under drought stress in *B. napus*.

### 2.2. Changes in Crystal Morphology, Content and Composition of Epidermal Wax under Varying Degrees of Drought Stress in B. napus

**Figure 2:**
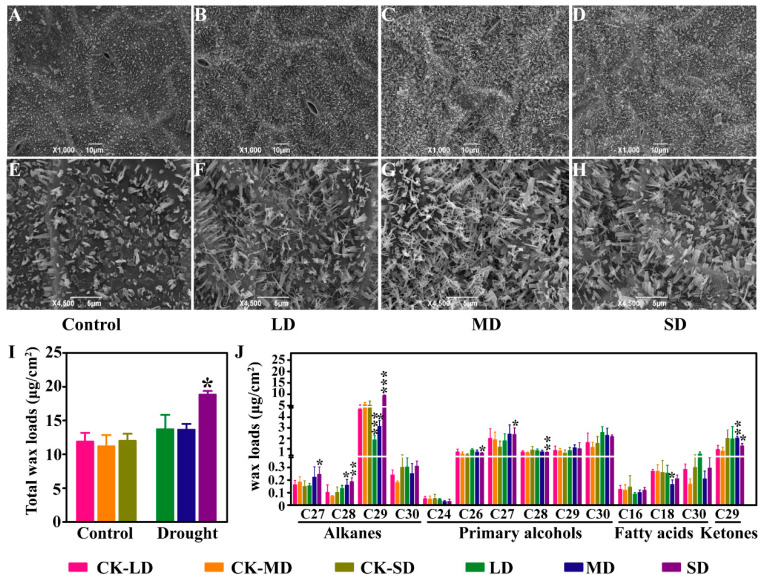
Morphology observation and determination of epidermal wax content and composition under varying degrees of drought stress. (**A**–**H**) Scanning electron microscope observation of epidermal wax morphology under varying degrees of drought stress at the magnifications of 1000× *g* (**A**–**D**) and 4500× *g* (**E**–**H**). (**I**) Determination of total wax content under varying degrees of drought stress. (**J**) Quantitative analysis of wax components by GC-MS. The data are the means ± SD (*n* = 4). LD, mild drought stress; MD, moderate drought stress; SD, severe drought stress; CK-LD, CK-MD, and CK-SD, the respective controls of LD, MD, and SD. Statistical significance was determined by Student’s *t*-test. * *p* < 0.05, ** *p* < 0.01, *** *p* < 0.001.

Except for water loss through stomata, non-stomatal transpiration determined by the cuticle is also an important way to control water loss. Epidermal wax is distributed on the surface of various plant organs and plays an important role in plant drought resistance. In order to assay the changes of epidermal wax morphology under different degrees of drought stress, the epidermal wax was observed by scanning electron microscopy (SEM). Our results showed that sparse plate-like and tube-like crystals were distributed on the leaf surface under the control (Figure 2A,E). With the increased degree of drought stress, the amount of epidermal wax crystals on the leaf surface increased significantly, and the shape of the epidermal wax crystals altered. The proportion of tube-like crystals was elevated under the LD and MD conditions (Figure 2B,C,F–G), while it was reduced under the SD conditions (Figure 2D,G).

In order to quantitatively study the changes of wax content, we further determined the total wax content and composition under varying degrees of drought stress using GC-MS. The results showed that the total wax loads have no significant differences between the control conditions but increased significantly under the SD conditions compared with the CK-SD conditions (Figure 2I). A total of four types of wax composition with different carbon chain lengths were identified, including alkanes with C27 to C30 chain lengths, primary alcohols with C24 to C30 chain lengths, fatty acids with C16, C18, and C30 chain lengths, and ketones with a C29 chain length. The contents of the alkanes with the C27, C28, and C29 chain lengths were increased by 66.8%, 83.7%, and 84.4% under the SD conditions compared with the CK-SD conditions, respectively, while the content of the alkanes with the C28 chain lengths was increased by 1.29 times under the MD conditions compared with the CK-MD conditions. However, the content of the alkanes with the C29 chain lengths was decreased by 42.8% and 61.2% under the LD and MD conditions compared with the CK-LD and CK-MD conditions, respectively. The content of the alkanes with C30 chain lengths did not significantly alter under drought stress. In addition, the primary alcohols are also one of the major epidermal wax components. The content of the primary with the C26 and C27 chain lengths was increased by 25.2% and 50.4% under the SD conditions compared with the CK-SD conditions, respectively, while the content of the primary with C28 was decreased by 25.7% under the SD conditions compared with the CK-SD conditions. For fatty acids, only the content of the C18 fatty acid was decreased by 39.0% under the MD conditions compared with the CK-MD conditions. The content of the C29 ketone, which was the only ketone identified in the epidermal wax, was increased by 1.55 times under the MD conditions compared with the CK-MD conditions but was decreased by 0.36 times under the SD conditions and the CK-SD conditions (Figure 2J). These results suggest that the morphology, content, and composition of epidermal wax are significantly altered under different degrees of drought stress in *B. napus*.

### 2.3. Changes in Cutin Content and Composition under Varying Degrees of Drought Stress in B. napus

**Figure 3:**
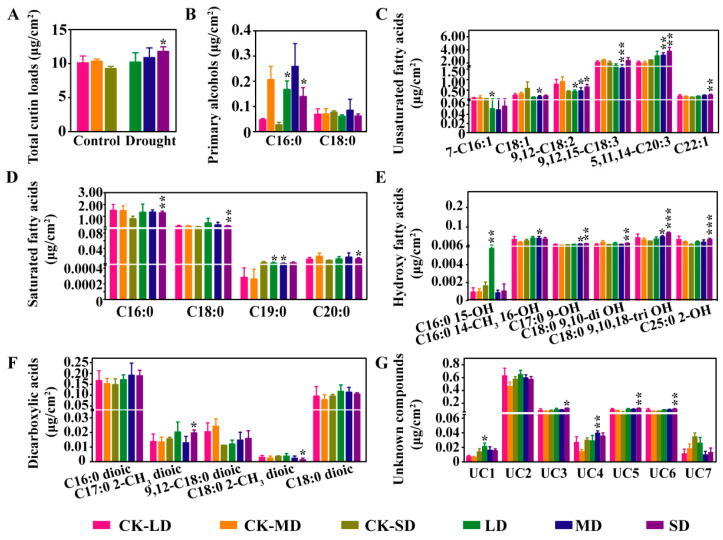
Quantitative analysis of cutin components by GC-MS under varying degrees of drought stress conditions. (**A**) Determination of total cutin content under varying degrees of drought stress. (**B**–**G**) Determination of primary alcohol content (**B**), unsaturated fatty acid content (**C**), saturated fatty acid content (**D**), hydroxy fatty acid content (**E**), dicarboxylic acids content (**F**), and unknown compounds content (**G**) under varying degrees of drought stress. LD, mild drought stress; MD, moderate drought stress; SD, severe drought stress; CK-LD, CK-MD and CK-SD, the respective controls of LD, MD, and SD. Statistical significance was determined by Student’s *t*-test. * *p* < 0.05, ** *p* < 0.01, *** *p* < 0.001.

Cutin also plays an essential role in cuticle transpiration. To investigate the alteration of cutin under varying degrees of drought stress, the total content and the composition of cutin were determined using GC-MS. The total cutin content increased by 27.3% under the SD conditions compared with the CK-SD conditions, while there was no significant difference under the LD and MD conditions compared with the CK-LD and CK-MD conditions, respectively (Figure 3A). A total of six types of cutin monomer composition with different carbon chain lengths were identified in the leaves, including primary alcohols with C16 and C18 chain lengths, unsaturated fatty acids with C16, C18, C20, and C22 chain lengths, saturated fatty acids with C16 to C20 chain lengths, hydroxy fatty acids with C16 to C18 and C25 chain lengths, dicarboxylic acids with C16 to C18 chain lengths, and seven types of unknown compounds (Figure 3B–G). The content of the C16:0 primary alcohols was significantly increased under the LD and SD conditions compared with the CK-LD and CK-SD conditions, respectively, while the content of the C18:0 primary alcohols did not significantly change under varying degrees of drought stress compared with the respective controls (Figure 3B). The content of the 7-C16:1 and 9,12-C18:2 unsaturated fatty acids was significantly decreased under the LD conditions compared with the CK-LD conditions, and the content of the C18:1, 9,12-C18:2, and 9,12,15-C18:3 unsaturated fatty acids was significantly decreased, whereas the content of the 5,11,14-C20:3 unsaturated fatty acid was significantly increased under the MD conditions. Under the SD conditions, the content of the 9,12-C18:2, 5,11,14-C20:3, and C22:1 unsaturated fatty acids was significantly elevated (Figure 3C). The content of the C19:0 saturated fatty acid was significantly increased under both the LD and the MD conditions, and the content of the C16:0, C18:0, and C20:0 saturated fatty acids was significantly increased only under the SD conditions (Figure 3D). The content of C16:0 15-OH hydroxyl fatty acid was significantly elevated under the LD conditions, and the other identified hydroxyl fatty acids were significantly elevated under both the MD and the SD stress conditions (Figure 3E). The C17:0 2-CH_3_ dicarboxylic acid content was significantly increased, whereas the C17:0 2-CH_3_ dicarboxylic acid content was significantly decreased under the SD conditions (Figure 3F). In addition, the content of some unknown compounds was also found to be significantly increased under drought stress (Figure 3G). Our results indicate that the total content of cutin increases under SD conditions, and the cutin monomer composition is severely affected under varying degrees of drought stress.

### 2.4. Transcriptome Analysis Revealed Gene Expression Alternation under Drought Stress

**Figure 4:**
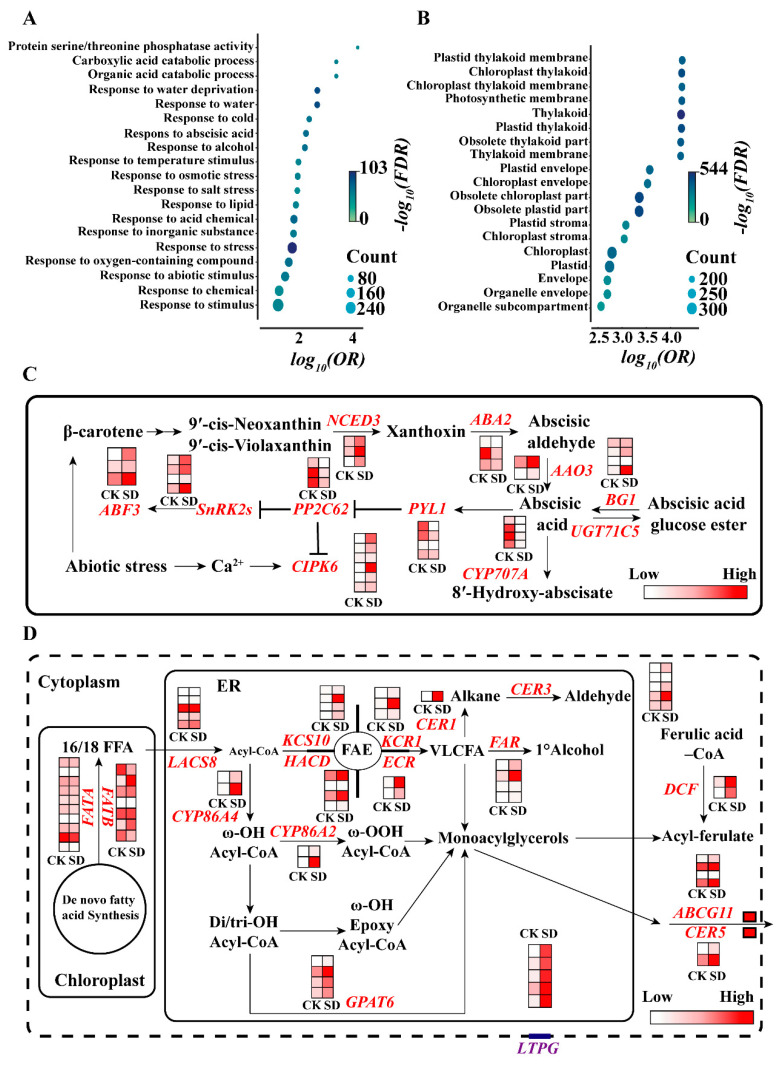
Transcriptome analysis of *B. napus* plants subjected to severe drought stress. (**A**) Gene ontology (GO) analysis of up-regulated differentially expressed genes (DEGs) under severe drought stress. (**B**) GO analysis of down-regulated DEGs under severe drought stress. (**C**) DEGs) involved in ABA biosynthesis and signal transduction pathways. (**D**) DEGs involved in the epidermal wax and cutin biosynthesis pathway. CK, control; SD, severe drought.

Considering these significant variations in the physio-biochemical traits under SD conditions, we performed the comparative transcriptome analysis to investigate changes in the gene expression between the control and the SD conditions (Table S1). A total of 1845 significantly differentially expressed genes (DEGs) were identified, of which 1050 were down-regulated genes and 795 were up-regulated genes (Figure S1A, Tables S2 and S3). In order to identify the most significantly influenced drought-related pathways, we performed the gene ontology (GO) enrichment analysis of the DEGs (Tables S4 and S5). The top 20 most significantly enriched GO terms of the up-regulated DEGs were mainly enriched in the response to water deprivation, abscisic acid (ABA), osmotic stress, and other abiotic stimuli and lipids (Figure 4A, Table S4). In addition, the top 20 most significantly enriched gene GO terms of the down-regulated DEGs were mainly enriched in photosynthesis-related pathways, such as the plastid thylakoid membrane, photosynthetic membrane, chloroplast stroma, chloroplast envelope, and chloroplast (Figure 4B, Table S5). In addition, we also performed KEGG analysis of the up-regulated and down-regulated DEGs (Tables S6 and S7). The results showed that these up-regulated genes were enriched in cutin, suberine, and wax biosynthesis pathways, plant hormone signal transduction, fatty acid degradation, secondary metabolites biosynthesis, and metabolic pathways (Figure S1B, Table S6), and these down-regulated genes were enriched in some biochemical processes, such as photosynthesis, and some metabolic pathways, such as porphyrin and chlorophyll metabolism, carbon metabolism, and nitrogen metabolism (Figure S1C, Table S7). Taken together, these results suggest that *B. napus* plants resist drought stress through various physio-biochemical processes, and photosynthesis will bear the brunt when the plants suffer from severe drought stress.

In order to better understand the cause of the changes in the physio-biochemical processes responding to drought stress in *B. napus*, we further analyzed the expression levels of the representative genes involved in ABA biosynthesis and the signal transduction pathways and the cuticle biosynthesis pathway. The expression of the ABA biosynthesis-related genes, such as *9-cis-epoxycarotenoid dioxygenase 3* (*NCED3*), *abscisic acid deficient 2* (*ABA2*), and *abscisic aldehyde oxidase 3* (*AAO3*), was significantly increased under the SD conditions. The expression of *β-glucosidase 1* (*BG1*), which hydrolyzes ABA glycosides to produce ABA, was significantly increased; conversely, the expression of *CYP707A* encoding an ABA 8′-hydroxylase involved in ABA catabolism was significantly decreased under the SD conditions (Figure 4C). For the ABA signaling transduction, the SD treatment significantly decreased the expression of some early signaling components, such as *PYR1* and *PP2C62* and significantly increased the expression of *SnRK2.2*. In addition, the expression of the downstream signaling components, such as *CIPK6* encoding a calcineurin B-like protein (CBL)-interacting protein kinase (CIPK) and *ABF3* encoding an ABA-responsive element binding factor, was also significantly increased in responding to SD. Moreover, the expression of the key genes involved in cuticle biosynthesis was also significantly altered under the SD conditions. The expression levels of *long-chain acyl-CoA synthetase 8* (*LACS8*), *ketoacyl-CoA synthase 10* (*KCS10*), *ketoacyl-CoA reductase 1* (*KCR1*), *hydroxyacyl-CoA dehydrase* (*HACD*), *enoyl-CoA reductase* (*ECR*), and *ECERIFERUM 3* (*CER3*) involved in epidermal wax biosynthesis and cytochrome P450 (*CYP*) *86A*, *CYP77A6*, *CYP77A4*, and *glycerol-3-phosphate acyltransferase 6* (*GPAT6*) involved in cutin biosynthesis were significantly increased under the SD conditions. The expression levels of the key genes involved in cuticle monomer transport, such as *CER5*, *ATP-binding cassette transporter G subfamily 11* (*ABCG11*), and *GPI-anchored lipid transfer protein1* (*LTPG1*) were also significantly increased (Figure 4D). Moreover, some up-regulated DEGs, such as *deficient in cutin ferulate* (*DCF*), *CYP86A2, cystathionine beta-synthase protein* (*CBS*)*, nitrile specifier protein 5* (*NSP5*)*, expansin-like B1* (*EXLB1*)*,* and *dehydrin family protein* (*RAB18*), and down-regulated DEGs, such as *dihydropyrimidine dehydrogenase* (*PYD1*), *abscisic acid-insensitive 2* (*ABI2*), *chloroplast unusual positioning 1* (*CHUP1*), *acyl activating enzyme 3* (*AAE3*), and *glc-hypersensitive mutant 2* (*GSM2*), were also found; their functions in relation to drought are still poorly understood in *B. napus* (Table S1). To validate the results of the transcriptome analysis, qRT-PCR analysis was performed to assay the expression levels of some DEGs under the SD conditions. We found that the expression levels of the ABA biosynthesis-related gene *NCED3*, the ABA metabolism-related genes *BG1*, the ABA signaling-related gene *CIPK6*, and the cuticle biosynthesis related genes, such as *CER3, LTPG1, LACS8, GPAT6, CYP86A2, KCS10*, *KCR1*, and *ECR*, were significantly higher under the SD conditions than those under the control. In contrast, the expression levels of the ABA signaling-related genes such as *PYL1* and *PP2C62* and the ABA metabolism-related gene *CYP707A* were significantly lower under the SD conditions than those under the control (Figure S2). These results suggest that ABA metabolism, ABA signaling, cuticle biosynthesis, and cuticle monomer transport, which contribute to physio-biochemical traits, play vital roles in adaptation to drought stress in *B. napus*.

### 2.5. Over-expression of BnaA01.CIPK6 Confers Drought Tolerance in B. napus

**Figure 5:**
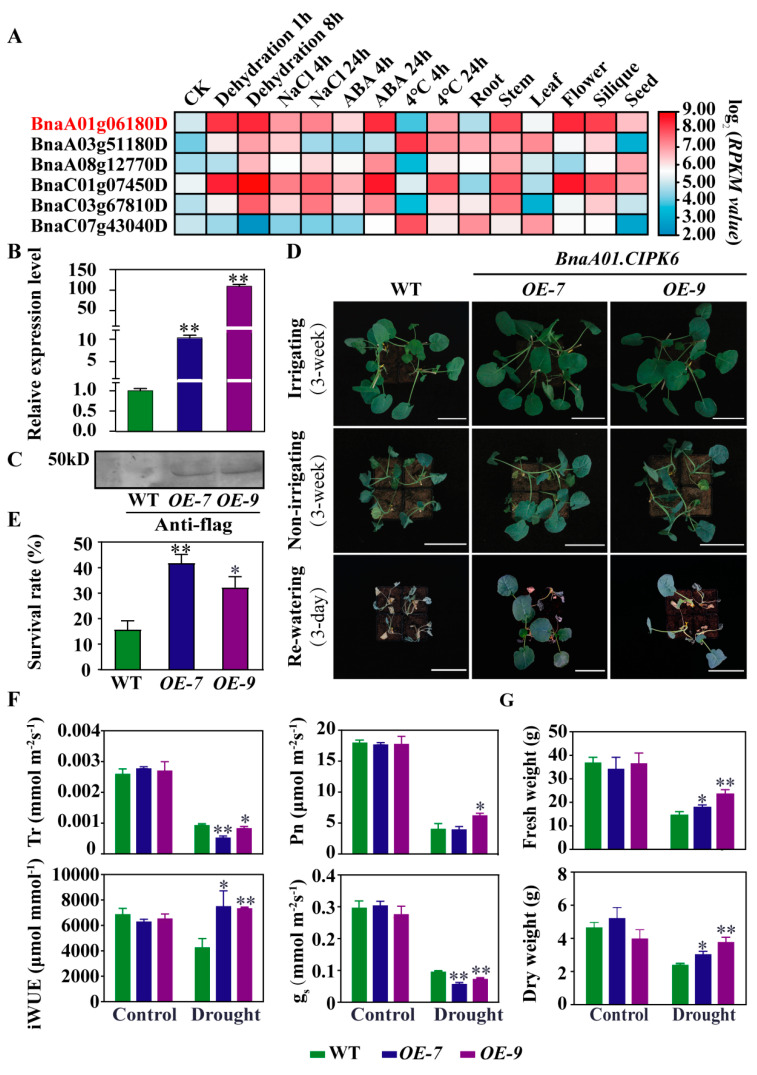
Over-expression of *BnaA01.CIPK6* confers drought tolerance in *B. napus*. (**A**) Expression pattern analysis of *BnCIPK6.* (**B**) Relative expression level of *BnaA01.CIPK6* gene in two *BnaA01.CIPK6-*overexpression lines, *OE-7* and *OE-9*. (**C**) Western blot analysis of *BnaA01.CIPK6* gene expression in *BnaA01.CIPK6-*overexpression (*OE-7* and *OE-9*). (**D**) The response of *BnaA01.CIPK6-*overexpression plants (*OE-7* and *OE-9*) to drought stress. Bar = 10 cm. (**E**) Survival rate of *BnaA01.CIPK6-*overexpression lines (*OE-7* and *OE-9*) under drought stress. (**F**) Measurement of photosynthesis-related parameters of *BnaA01.CIPK6-*overexpression lines (*OE-7* and *OE-9*) under drought stress. (**G**) Aboveground biomass of *BnaA01.CIPK6-*overexpression lines (*OE-7* and *OE-9*) under drought stress. The data are the means ± standard deviation (*n* = 4). Statistical significance was assayed by Student’s *t*-test. * *p* < 0.05, ** *p* < 0.01.

*BnaA01.CIPK6* was identified as an up-regulated DEG in responding to SD (Figure 4C), and we analyzed the expression levels of *BnaA01.CIPK6* under various abiotic stresses and the expression pattern of *BnaA01.CIPK6* in various tissues of *B. napus*. The results showed that the expression levels of *BnaA01.CIPK6* were significantly increased under the dehydration, NaCl, ABA, and 4 °C treatments, and *BnaA01.CIPK6* was expressed in the root, stem, leaf, flower, silique, and seed of *B. napus* (Figure 5A). To further study the function of *BnaA01.CIPK6* under drought stress, overexpression lines of *BnaA01.CIPK6* were generated, and two lines, *OE-7* and *OE-9*, in which the expression of *BnaA01.CIPK6* was increased by 10.12 and 109.40 times, respectively, were selected for further study (Figure 5B). The *BnaA01.CIPK6* protein was detected by a flag antibody using Western blot analysis, and the protein size was similar to the predicted size of ~46 kD obtained from the Uniprot database (https://www.uniprot.org/ accessed on 28 June 2022) (Figure 5C). The results showed that the survival rate was significantly increased in the overexpression lines of *BnaA01.CIPK6* (*OE-7* and *OE-9*) under drought stress compared with that of WT (Figure 5D,E). Moreover, these two overexpression lines also exhibited significantly lower stomatal conductance (g_s_) and transpiration rate (Tr) and significantly higher water use efficiency (iWUE) than that of WT, and the line *OE-9* with a higher level of *BnaA01.CIPK6* expression exhibited a significantly increased net photosynthetic rate compared with WT under drought stress (Figure 5F), which may lead to a significant increase in the fresh weight and dry weight under drought stress (Figure 5G). These results suggest that overexpression of *BnaA01.CIPK6* enhances drought resistance in *B. napus*.

## 3. Discussion

Drought stress adversely affects plant growth and development then significantly reduces crop productivity and yields [29]. Drought resistance is a complex trait depending on the severity of the drought and the stress duration, as well as the plant developmental stage. Plants have developed a wide variety of mechanisms to cope with drought stress through growth and physio-biochemical responses. Although a great number of studies have focused on the resistance to drought stress, there is little knowledge on the response to varying degrees of drought stress in *B. napus*. In this study, we characterized the physio-biochemical traits in response to varying degrees of drought stress in *B. napus* (Figure 1A). Transcriptome analysis revealed that ABA biosynthesis, metabolism, and signaling transduction and cuticle biosynthesis play a vital role in responding to drought stress, and drought stress caused a powerful influence on photosynthesis in *B. napus* (Figure 4C,D).

Stomatal transpiration and cuticular transpiration are the two main ways of plant water loss. Stomatal transpiration is mainly regulated by stomatal movement, size, and density [30]. ABA can induce stomatal closure, which inhibits water loss and photosynthesis and then influences yield [31]. Previous research has also shown that drought stress can induce ABA biosynthesis in plants [32]. Our results showed that ABA content increased significantly under varying degrees of drought stress in *B. napus* (Figure 1D), which was consistent with the results from previous studies [33,34]. Stomatal closure caused by ABA directly inhibited the efficiency of photosynthesis under drought stress [35]. Our results showed that ABA contents under varying degrees of drought stress conditions were significantly higher than those under the control (Figure 1D), which may result in stomatal closure, thereby leading to the lower stomatal conductance, transpiration rate, and net photosynthetic rate. Interestingly, GO and KEGG analysis also revealed that the photosynthesis pathway was severely affected under drought stress (Figure 4B and Figure S1B). Photosynthesis, which is sensitive to drought stress, is closely correlated to biomass production [36]. Drought stress reduces stomatal conductance, and thus, carbon assimilation and biomass production are decreased [37]. Therefore, biomass is one of the important indicators of drought stress responses. Previous studies have reported that drought durations largely affect the biomass and are positively associated with yield [38,39]. Our results also showed that both the fresh weight and the dry weight exhibited a declined trend with the increase in drought degree (Figure 1B,C).

The results from the transcriptome analysis showed that the expression levels of ABA biosynthesis-related genes such as *NCED3* and *AAO3* were significantly increased, while the expression levels of ABA metabolism-related genes such as *CYP707A2* and *CYP707A4* (Figure 4C) were significantly decreased, which was consistent with the elevated ABA content under drought stress (Figure 1D). In addition, the expression of *PYL1*, *ABF3, ABI2,* and *SnRK2.10* involved in ABA signal transduction was in response to drought stress. These results were consistent with previous studies [40,41,42,43,44]. Calcium as a nutrient is necessary for plant normal growth and development, and it also acts as a secondary messenger mediating ABA signals in plant responses to environmental stimuli. The CBL-CIPK complex was first identified in a genetic screen for a *salt oversensitivity* (*SOS*) phenotype. *SOS2* (*CIPK24*) interacts with *CBL4* (*SOS3*), which in turn activates the plasma membrane-localized Na^+^/H^+^ antiporter (*SOS1*) and vacuolar H^+^-ATPase under salt stress. The *CBL*-*CIPK* complex involved in signaling networks plays crucial roles in plant responses to environmental stimuli. A total of seven *CBL* and twenty-three *CIPK* genes were identified in *B. napus* [45]. Previous research has shown that the overexpression of *AtCIPK6* confers the salt and drought tolerance in Arabidopsis [46,47,48]. Our results indicated that the expression of the *BnaA01.CIPK6* gene was induced by drought stress (Figure 5A) and that *BnaA01.CIPK6* positively regulates drought resistance in *B. napus* (Figure 5B–G).

Cuticle transpiration also plays an important role in plant water loss. The epidermal wax biosynthesis with the change of epidermal wax morphology can be induced by drought stress [49,50,51]. Our results showed that the plate-like and tube-like wax crystals were observed on the leaf surface of *B. napus*, showing a similar polymorphism of epidermal wax morphology to *B.oleracea* L. [52]. Interestingly, drought stress was found to increase the proportion of tube-like crystals in *B. napus* (Figure 2E–H). Except for the changes of morphology, drought stress increases the content of epidermal wax and alters the composition in Arabidopsis, rice, wheat, and maize [53,54,55,56]. Under drought stress, the total content of wax on the leaves increased by 75%, which is mainly due to the increased content of the waxy alkane [53]. Although the content of each component varies differently, our results also showed that the total amount of epidermal wax was increased under SD conditions (Figure 2I,J). The content of the C27, C28, and C29 alkanes and the C26 and C27 primary alcohols in epidermal wax was significantly elevated under SD conditions compared with the control (Figure 2J), which is consistent with the previous studies. However, the content of the C29 alkane was significantly decreased under the LD and MD conditions for unknown reasons. According to the transcriptome data, the expression levels of several key enzymes related to the biosynthesis of very long-chain fatty acids (VLCFAs), such as *KCS10, KCR1, ECR,* and *HACD*, were up-regulated under the SD conditions. Moreover, the expression levels of the key genes *CER1*, *CER3*, and *FAR*, which are responsible for the biosynthesis of alkanes, aldehydes, and alcohols, respectively, were also found to be up-regulated under drought stress (Figure 4D). These results were consistent with the changes in content and composition of epidermal wax in *B. napus*.

Cutin, the framework of the plant cuticle, is a polyester composed of oxygenated fatty acids cross-linked by ester bonds. A previous study has demonstrated that the content of the total cutin content is increased under water deficit conditions in Arabidopsis [53]. The total content of cutin increased under the SD conditions (Figure 3A). Moreover, the content of most of the components in cutin, such as saturated fatty acids, hydroxy fatty acids, and unknown components, increased; however, the content of some components, such as unsaturated fatty acids C16:1, C18:1, C18:2, and C18:3, decreased under varying degrees of drought stress conditions. A great number of up-regulated genes were identified to be related to cutin biosynthesis under drought stress, such as *LACS8* encoding a long-chain acyl-CoA synthetase, *CYP86A4* and *CYP86A2* encoding the synthesis of ω-OH Acyl-CoA and ω-OOH Acyl-CoA (Figure 4D), which have been reported to produce the long-chain acyl-CoA and hydroxy fatty acid components of cutin [57]. These results were consistent with the alteration in content and composition of cutin in *B. napus*. Taken together, the changes in content and composition of epidermal wax and cutin may contribute to the variation in physio-biochemical traits by regulating cuticle transpiration under drought stress in *B. napus*.

Many genes reported previously in drought stress responses, such as *BnaC04g45800D* (*BnLTP1*), *BnaAnng01320D* (*BnDREB2A*), *BnaC08g07490D* (*BnLEA1*)*, BnaC03g12050D* (*BnLTP3*)*,* and *BnaC02g45160D* (*BnRAB18*) [58,59,60], were identified. Additionally, some un-reported drought-related genes, such as *BnaA06g10010D* (*BnCBS*) encoding a cystathionine beta-synthase and *BnaC02g38400D* (*BnNSP5*) encoding a nitrile specifier protein, were also identified under drought stress in *B. napus*. (Table S1). These findings provide a basis for understanding the molecular mechanisms underlying drought tolerance in *B. napus*, and the molecular mechanism of the *BnaA01.CIPK6* gene and the function of unreported genes in response to drought stress need to be further studied in the future.

## 4. Materials and Methods

### 4.1. Plant Materials and Drought Stress Treatments

Self-seeds of *B. napus* cultivar CHUYOU1 were harvested from experimental fields in Huazhong Agricultural University. *B. napus* plants were grown in a ventilated and rain-sheltered growth room, and the temperatures were kept within 10 °C (dark phase) and 24 °C (light phase) during the entire growth period. Relative air humidity was kept at least 65%, and the plants were kept on a 12 h light/dark cycle. Seedlings at the four-to-five-leaf stage were treated with mild drought stress (50% relative water content, LD), moderate drought stress (40% relative water content, MD), and severe drought stress (30% relative water content, SD), respectively. The respective controls under normal conditions (80% relative water content, CK-LD, CK-MD, and CK-SD) were set up for these different drought conditions to eliminate the effects of plant development. The sampling times were 17–18 November 2020 (CK-LD and LD), 28–29 November 2020 (CK-MD and MD), and 6–7 December 2020 (CK-SD and SD), respectively, for RNA extraction and determination of biomass, ABA, and wax and cutin contents. The calculation formula for the experiment on the soil relative water content in pots is as follows.

(1) $$\mathrm{Field}\;\mathrm{capacity}=\frac{\left(W2-W0\right)-\left(W1-W0\right)}{\left(W2-W0\right)}\;\times \;100\%\;$$

(2) $$\mathrm{Soil}\;\mathrm{relative}\;\mathrm{water}\;\mathrm{content}=\frac{\left(W3-W0\right)-\left(W1-W0\right)}{\left(W3-W0\right)*\mathrm{Field}\;\mathrm{capacity}}\;\times \;100\%$$

W0: The weight of an empty pot

W1: The weight of the pot and dry soil

W2: the weight of the pot full of watered soil

W3: the weight of pot under drought stress

### 4.2. Determination of ABA Content

Approximately 50–100 mg leaf sample was ground and added to 1 mL of extraction solution containing 2-propanol/water/concentrated HCl (2/1/0.002, *v*/*v*/*v*) and vortexed for 30 min at 4 °C. Then, 1 mL methylene chloride (CH_2_Cl_2_) was added and vortexed for 30 min at 4 °C. After centrifugation at 5000× *g* for 10 min at 4 °C, the lower layer (around 1 mL) was transferred to a new tube and dried under a stream of nitrogen. The dried samples were dissolved in 200 µL of solving liquid containing methanol/0.05% formic acid (1/1, *v*/*v*) and then were filtered through a 0.45 μm membrane. The filtered samples were transferred to sample vials for analysis in a HPLC-MS/MS system (QTRAP^®^ 6500+ LC-MS/MS System, SCIEX) consisting of HPLC and QTRAP 6500.

### 4.3. RNA-Seq Alignment and Differential Expression Analysis

Total RNA was extracted from leaf samples under CK-SD and SD conditions by RNA prep Pure Plant Kit (TIANGEN, Beijing, China). Transcriptome sequencing of the samples was performed at the Novogene Bioinformatics Institute (Novogene, Beijing, China). The low-quality adapter sequences were filtered with Trimmomatic to obtain the clean data. The software Bowtie2 v2.2.9 was used to map to the reference genome, and the gene expressions were calculated by RSEM v1.3.0. Differentially expressed genes were identified by DEseq2 (|log2FoldChange| > 1 and padj < 0.05). The differentially expressed genes were performed with Gene Ontology (GO) (http://www.geneontology.org/ accessed on 28 June 2022) and KEGG analysis (KEGG: http://www.genome.jp/kegg/ accessed on 28 June 2022), respectively.

### 4.4. Quantitative Real-Time PCR Analysis

To validate the RNA-seq data, some DEGs were selected for qRT-PCR assay with three biological replicates. All primers were designed using PRIMER5 software, and the sequences were listed in Table S1. The RNA samples used for qRT-PCR analyses were the same ones used in the RNA-Seq. RNA from each sample was reverse transcribed using the EasyScript^®^ One-Step gDNA Removal and cDNA Synthesis SuperMix (Beijing Transgen Biotech Co. Ltd., Beijing, China). Quantitative real-time PCR (qRT-PCR) was performed using the Perfect Start TM Green qPCR Super Mix (Beijing Transgen Biotech Co. Ltd., Beijing, China) with the CFX Connect TM Real-Time System (Bio-Rad Laboratories, Inc., CA, USA).

### 4.5. Scanning Electron Microscopy

The samples were placed between two pieces of filter paper and were thoroughly dried at 30 °C. Approximately 25 mm^2^ of dried samples was mounted on aluminum stubs using double-sided adhesive tape and then coated with gold particles using 90 s bursts from a sputter coater. The wax crystal morphology was observed with a field emission SEM (JSM-6390/LV, JEOL, Tokyo, Japan) device using a 10 kV accelerating voltage and an 8.4 mm working distance.

### 4.6. Photosynthetic Parameter Measurements

Photosynthetic parameter measurements were performed on the youngest fully expanded leaves at the seedling stage using the Li-Cor 6800 portable photosynthesis system (Li-Cor Bioscience) equipped with the Red/Blue (9:1) light source (Li-Cor Part No. 6400-2B, area 6 cm^2^). The data were saved after the measured parameters remained stable and under the following conditions: ambient RH = 50–80%, 400 μmol mol^−1^ CO_2_, 1200 μmol m^−2^s^−1^ photosynthetically active radiation, 25 °C leaf temperature, 500 μmol s^−1^ flow rate, and 10,000 rpm fan speed. The measured Pn and Tr were used to calculate iWUE, and the calculation formula was as follows.

iWUE= Pn /Tr

### 4.7. Determination of Leaf Epicuticular Wax

Before wax extraction, the surface area of samples were calculated using Image J software. About 30 cm^2^ of leaves was put in 10 mL chloroform, containing a 10 mg n-tetracosane (C24) internal standard and shaken at room temperature for 30 s. The chloroform solution was transferred to a new glass tube and evaporated under a gentle stream of nitrogen. After drying, 1 mL of chloroform was added to dissolve the samples which were then transferred to the sample vials for analysis in a GC-MS system (Shimadzu GCMS-QP2010). Prior to GC-MS analysis, the dissolved samples were converted to the trimethylsilyl derivatives with 20 mL of bis-(N, N-trimethylsilyl)-tri-fluoroacetamide (BSTFA) for 1 h at 70 °C. GC-MS was used to determine the composition and content of the epicuticular wax.

### 4.8. Determination of Leaf Cutin

The leaf samples were transferred to a glass test tube and immersed in a mixture of methanol/chloroform (1/1, *v*/*v*) and 5% butylated hydroxytoluene (BHT, the final concentration was 0.01%). After being shaken at 37 °C for 2 h, the extraction mixture was collected. This step was repeated 3 times until the color of the leaves changed from green to colorless. The mixture was poured out and dried thoroughly at 35 °C. A 9 mL mixture composed of methanol/concentrated sulfuric acid/chloroform (10/0.5/1, *v*/*v*/*v*) and 5% BHT (the final concentration was 0.01%) was added to the samples. Then, 10 μg methyl heptadecanoate was added as an internal standard. The samples then were placed in an 80 °C water bath for 2 h and were shaken every 20 min with tightly capped bottle caps. After cooling, 4.5 mL of 0.9% NaCl was added, and then, 6 mL of a mixture containing n-hexane and chloroform at a ratio of 4:1 (*v*/*v*) was added to the samples and shaken for 30 s. After centrifuging at 2000 r/min for 1 min, the supernatants were carefully transferred to the new glass test tubes with a glass straw. Then, 3 mL of 0.9% NaCl was added to supernatants in the glass test tubes, which were shaken for 30 s and centrifuged at 2000 r/min for 1 min, and then, the supernatants were transferred to new glass test tubes again. After being dried under a stream of nitrogen, the samples were dissolved in 0.5 mL chloroform and placed in a refrigerator at −20 °C. The silanization reactions was same as the methods used in the determination of the leaf epicuticular wax. GC-MS was used to determine the composition and content of the cutin.

### 4.9. Vector Construction and Genetic Transformation

In order to clone *BnaA01.CIPK6,* primers were designed according to CDS sequences from the *B. napus* genome. The primers used for cloning are listed in Table S1. The amplified products were digested and cloned into the pCAMBIA1300s, a plant binary vector with a flag tag for overexpression vector construction. The *BnaA01. CIPK6-*overexpression vector was transformed into Agrobacterium tumefaciens GV3101, which was used to infect the hypocotyl of *B. napus* for genetic transformation. The details of the methods of genetic transformation can be found in [61].

### 4.10. Western Blot Analysis

The leaves were ground to powder in liquid nitrogen and transferred to a 1.5 mL centrifuge tube. After adding 200 μL extraction buffer (100 mM Tris-HCl, 150 mM NaCl, 2.5 mM EDTA, 2.5% SDS) and incubating at room temperature, the samples were then centrifuged for 15 min at 12,000× rpm. The supernatants (40 mL) were mixed with 10 mL 5×SDS-loading buffer for sample preparation. The proteins were separated by SDS-polyacrylamide gel electro phoresis and transferred to polyvinylidene difluoride (PVDF) membrane via electroblotting. The membranes were blocked in 5% BSA for 1 h and incubated overnight at 4 °C with the primary antibodies rabbit anti-Flag Tag (1:2000; Sigma SAB4301135). The membranes were then probed with secondary antibodies goat anti-rabbit (1:2000; Sigma A3687) at room temperature for 2.5 h. The signals were revealed by incubation in 5-bromo-4-chloro-3-indolyl phosphate (BCIP)/nitro blue tetrazolium (NBT) solution (Sigma B5655).

## 5. Conclusions

The characterization of multiple physio-biochemical traits and comparative transcriptome analysis proved to be powerful strategies for analyzing the response of *B. napus* to varying degrees of drought stress. The changes in fresh weight, dry weight, ABA content, photosynthesis-related parameters, epidermal wax crystal morphology, and the content and composition of wax/cutin were detected under different degrees of drought stress. Furthermore, a total of 1845 DEGs were identified under severe drought stress. A large number of DEGs were involved in various biological processes such as photosynthesis and stress responses. The discovery of these drought-responsive proteins lays a foundation for future studies directed toward functional characterization and increases the understanding of the molecular mechanisms underlying the regulatory network in drought stress resistance in *B. napus*.

## Supplementary Materials

The following supporting information can be downloaded at: https://www.mdpi.com/article/10.3390/ijms23158555/s1.
