# Supplementary material for: Combining Physio-Biochemical Characterization and Transcriptome Analysis Reveal the Responses to Varying Degrees of Drought Stress in Brassica napus L
Source: Int J Mol Sci. 2022 Aug 2;23(15):8555. doi: 10.3390/ijms23158555 (PMC9368929; doi:10.3390/ijms23158555)
Supplement: Supplementary file 1 [file ijms-23-08555-s001.zip › ijms-1816366 supplementary figures from text.pdf]

SUPPORTING INFORMATION

Figure S1 Distribution of differentially expressed genes (DEGs) and Kyoto Encyclopedia of Genes and Genomes (KEGG) pathway analysis.

Figure S2 Validation of RNA-seq data by qRT-PCR.

Table S1 Quantitative analysis of gene expression under CK-SD and SD conditions.

Table S2 Up-regulated differentially expressed genes.

Table S3 Down-regulated differentially expressed genes.

Table S4 GO categories of up-regulated DEGs.

Table S5 GO categories of down-regulated DEGs.

Table S6 KEGG enrichment analysis of up-regulated DEGs

Table S7 KEGG enrichment analysis of down-regulated DEGs

Table S8 Primers used in this study.

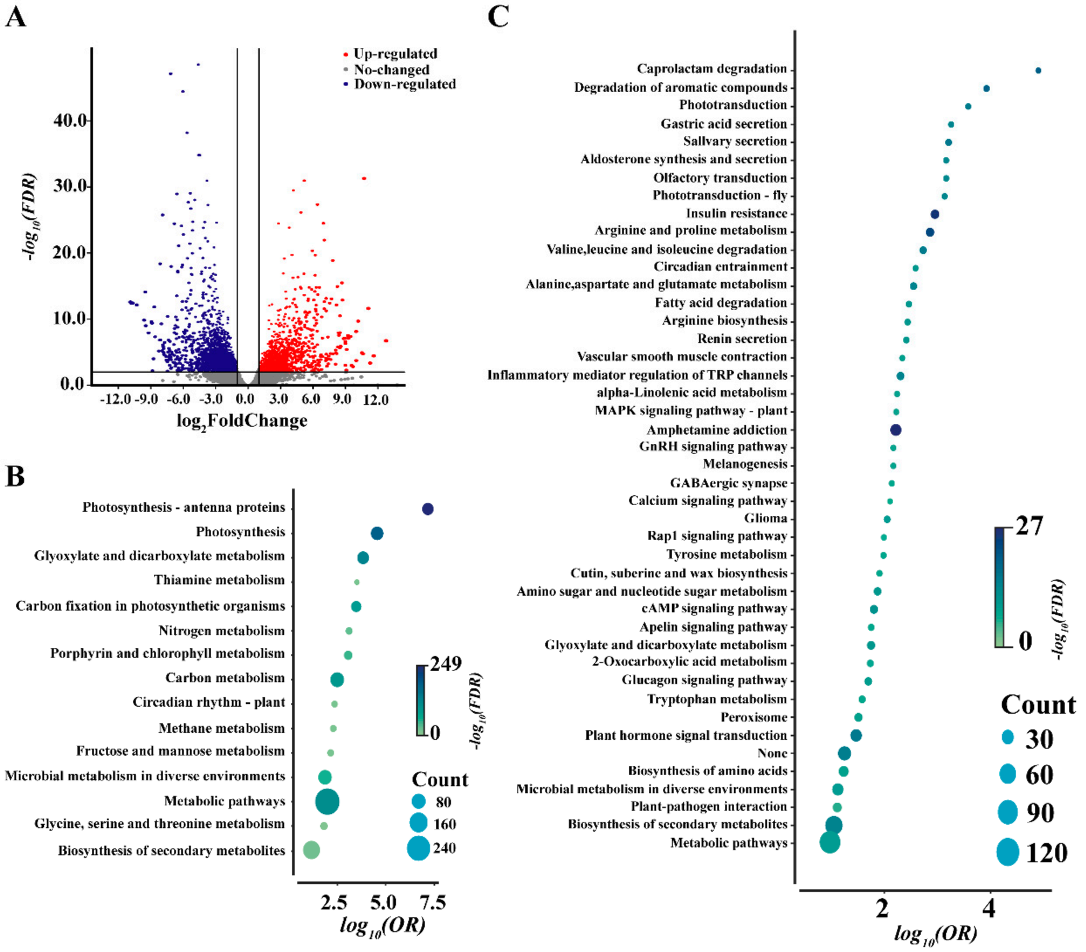

FIGURE S1 Distribution of differentially expressed genes (DEGs) and Kyoto Encyclopedia of Genes and Genomes (KEGG) pathway analysis. (A) Volcano plots showing the DEGs distribution between

normal and severe drought stress conditions. **(B)** KEGG pathway analysis of the up-regulated DEGs. **(C)** KEGG pathway analysis of the down-regulated DEGs.

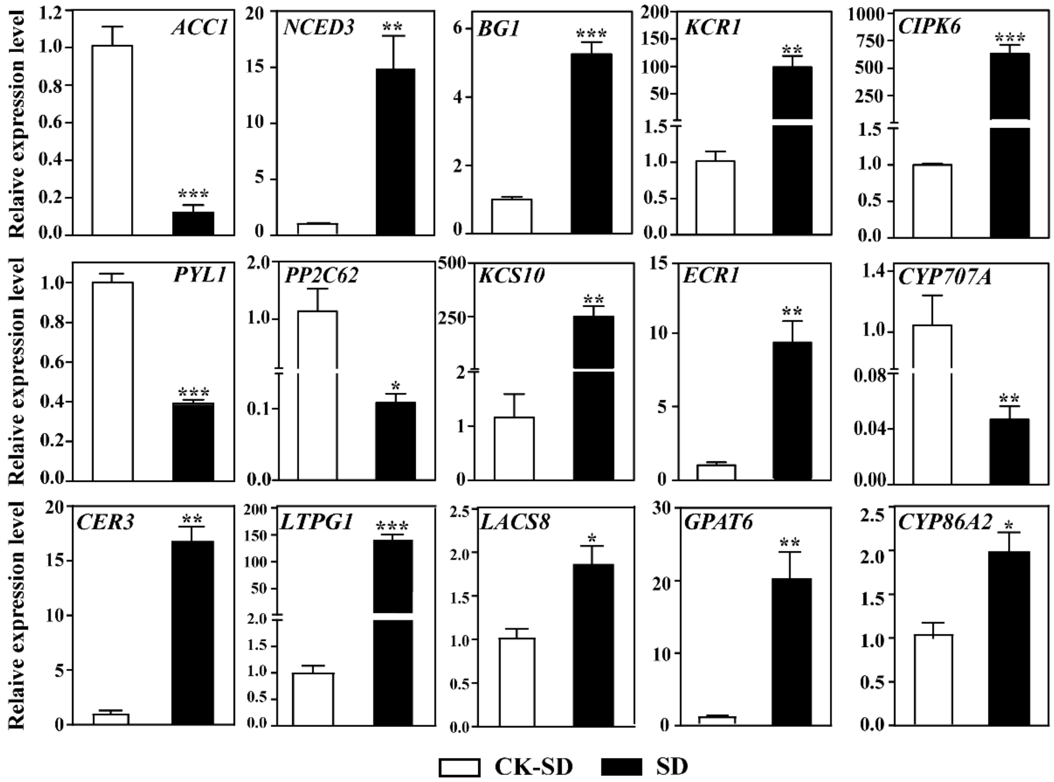

FIGURE S2 Validation of RNA-seq data by qRT-PCR. Acetyl-CoA-Carboxylase 1(*ACC1*); Nine-Cis-Epoxycarotenoid Dioxygenase 3(*NCED3*); β-Glucosidase 1(*BG1*); Ketoacyl-CoA Reductase 1 (*KCR1*); Calcineurin B-like Interacting Protein Kinase 6 (*CIPK6*); Pyrabactin resistance-Like 1(*PYL1*); clade-A type-2C protein phosphatases 62(*PP2C62*); Ketoacyl-CoA Synthase 10 (*KCS10*); Enoyl-CoA Reductase 1 (*ECR1*); *CYP707A* encode 8'-hydroxylase; ECERIFERUM 3 (*CER3*); GPI-anchored lipid transfer protein 1 (*LTPG1*); Long-chain Acyl-CoA Synthetase 8 (*LACS8*); Glycerol-3-phosphate acyltransferase 6 (*GPAT6*). Cytochromes P450 *CYP86A2*. The data are the means ± SD (n = 3). Statistical significance was determined by Student’s T-test. *p < 0.05, **p < 0.01, ***p < 0.001.
